# Representative boreal forest habitats in northern Europe, and a revised model for ecosystem management and biodiversity conservation

**DOI:** 10.1007/s13280-020-01444-3

**Published:** 2021-01-17

**Authors:** Håkan Berglund, Timo Kuuluvainen

**Affiliations:** 1 Swedish Species Information Center, Swedish University of Agricultural Sciences, Box 7007, 750 07 Uppsala, Sweden; 2 Department of Forest Sciences, University of Helsinki, Box 27, 00014 Helsinki, Finland

**Keywords:** Biodiversity conservation, Forest dynamics, Green infrastructure, Landscape management, Natural range of variation, Restoration, Sustainable forestry

## Abstract

The natural range of variation of ecosystems provides reference conditions for sustainable management and biodiversity conservation. We review how the understanding of natural reference conditions of boreal forests in northern Europe has changed from earlier perceptions of even-aged dynamics driven by stand-replacing disturbances towards current understanding highlighting the role of non-stand-replacing disturbances and the resultant complex forest dynamics and structures. We show how earlier views and conceptual models of forest disturbance dynamics, including the influential ASIO model, provide estimates of reference conditions that are outside the natural range of variation. Based on a research synthesis, we present a revised forest reference model incorporating the observed complexity of ecosystem dynamics and the prevalence of old forests. Finally, we outline a management model and demonstrate its use in forest ecosystem management and show how regional conservation area needs can be estimated. We conclude that attaining favourable conservation status in northern Europe’s boreal forests requires increasing emphasis on ecosystem management and conservation for old forest characteristics.

**Electronic supplementary material:**

The online version of this article (10.1007/s13280-020-01444-3) contains supplementary material, which is available to authorized users.

## Introduction

Current policies of sustainable forest management highlight the value of multiple ecosystem services and nature’s benefits to people (IPBES 2019). A crucial question is how to reconcile timber production, conservation of biodiversity, and other ecosystem services in human-dominated landscapes under increasing demands for wood and biomass (Hanski 2011; Felton et al. 2019; Angelstam et al. 2020). The natural (or historical) range of variation (NRV) of ecosystems is an important baseline for developing strategies for ecosystem management and biodiversity conservation (Landres et al. 1999). The rationale is that emulating and maintaining the representation of forest disturbance dynamics and structures similar to those found under natural circumstances (the coarse filter) are advantageous to biodiversity conservation and ecosystem resilience (Angelstam 1998; Bergeron et al. 2002; Johnstone et al. 2016). Furthermore, setting aside and aggregating biodiversity conservation areas that capture the full range of natural forest developmental stages are considered necessary for sustaining biodiversity, including specialized species requiring natural forest habitat (Hanski 2011; Angelstam et al. 2020).

Protecting representative habitat types and their various developmental stages is a common approach in biodiversity conservation (Angelstam and Andersson 2001; Lõhmus et al. 2004). In Europe, European Union (EU) Member States are required to take actions to achieve favourable conservation status for natural forest habitat types delineated by the Habitats Directive (DG Environment 2017). It involves measures for maintaining favourable reference areas, i.e. a certain total area considered the minimum necessary to ensure the long-term viability of the habitat types and their typical species in a given biogeographical region. Estimates of such large-scale area needs necessitate a sound understanding of natural disturbance regimes and their ecological impacts.

In northern Europe, the boreal forest is the key terrestrial ecosystem, but it has for most parts been strongly transformed due to a long history of intensive utilization and modern forestry based on even-aged management and clear-cut harvesting (Östlund et al. 1997; Linder and Östlund 1998). Particularly the proportion of old forests have decreased and been replaced by young, post-harvest forests (Kuuluvainen et al. 2015). This large-scale change may be further strengthened as a result of forestry intensification, but also due to future climate-induced changes in natural disturbance frequency and severity (Kuuluvainen and Gauthier 2018).

The conditions of boreal forests prior to intensive human usage are important as baselines for ecosystem management and biodiversity conservation. This refers to conditions where, while acknowledging that humans have to some degree probably been omnipresent in all boreal forests throughout history (Josefsson et al. 2010), human influence has been negligible and the natural forest dynamics and structure have prevailed (Brūmelis et al. 2011). Although historical records may infer the magnitude of past changes in forest conditions (e.g. Östlund et al. 1997; Linder and Östlund 1998; Axelsson and Östlund 2001), detailed survey data on forest dynamics and structure are generally lacking. An alternative approach is to reconstruct the reference conditions based on an understanding of natural ecosystem dynamics (Angelstam and Kuuluvainen 2004, Johnstone et al. 2016). In essence, conceptual models of natural forest dynamics are developed as a basis for formulating strategic targets for ecosystem management and biodiversity conservation (Angelstam 1998; Bergeron et al. 2002).

In this paper, we (1) first review earlier perceptions and current understanding of the natural dynamics and structure of boreal forests in northern Europe and (2) evaluate the adequacy and use of previous models, including the influential ASIO model (Angelstam 1998), for estimating reference conditions, guiding management and analysing conservation areas needs of these forests. Based on this evaluation, we (3) present a revised model of reference condition based on scientific understanding of boreal forest dynamics and structure. We then (4) outline an ecosystem management model for how the targeted reference conditions may be emulated. We also (5) show how conservation area needs of representative natural forests and their developmental stages may be estimated based on the revised reference model.

## Earlier perceptions

For decades, natural boreal forests were viewed as homogeneous, low-diversity ecosystems, the dynamics and structure of which were governed by stand-replacing disturbances with fairly short return intervals (around 50–100 years). According to this view (e.g. Mielikäinen and Hynynen 2003), disturbances particularly in the form of high-severity fires led to the development of even-aged, structurally homogeneous forest with development starting with newly disturbed areas and successively reaching an assumed stable state called ‘climax’ (Sirén 1955; Zackrisson 1977; Johnson 1992). This view of even-aged forest development after high-severity fires as the norm in boreal forest dynamics was founded on some early studies of northern European boreal forests (Ilvessalo 1927, 1937; Sirén 1955; Zackrisson 1977), but particularly on generalizations extrapolated from North American research (Johnson 1992; Payette 1992).

Based on this premise, the forest age-class distribution at large scales (such as a landscape or region) was modelled with deterministic time-since-fire probability distribution models, assuming that all forest stands were affected by the stand-replacing fires and with equal and constant probability of being affected over time. Forest age-class distribution models were Weibull or negative exponential probability distributions (Johnson 1992; Johnson and Gutsell 1994). The average return interval of fires is a key parameter in these models. For instance, with a negative exponential model, constant proportions of forests are predicted to be affected by disturbances with shorter (63.2%) or longer (36.8%) intervals than the average return interval. Thus, these models assuming stand-replacing disturbances with short return intervals (< 100 years) predict that young forests mainly dominate the forest age-class distribution, while old forests are estimated to be a minor component of the landscapes. For example, the negative exponential model predicts that 13.5% (exp^− 100/50^) of forests are older than 100 years if the average fire interval is 50 years.

The simplified view of stand-replacing, even-aged forest dynamics as the natural norm has been used as an influential argument in favour of compartment-wise, even-aged management systems in the boreal forest. This view has also been used in northern Europe to promote the use of intensive forestry based on clear-cutting as a nature-emulating method (Sirén 1955; Fries et al. 1997; Mielikäinen and Hynynen 2003). However, such simplified views cannot be justified in light of the current understanding concerning the intrinsic dynamics and structure of boreal forests in northern Europe. This observation has far-reaching consequences for forest ecosystem management and conservation.

## Current understanding

The earlier perceptions of natural forest dynamics and structure in the northern European boreal region have been largely revised due to new research evidence (summarized in Kuuluvainen 2009; Kuuluvainen et al. 2015). Two findings are particularly important from the sustainable management and conservation viewpoints. Firstly, in their natural dynamics, forests are shaped by more diverse and often non-stand-replacing disturbances than previously understood. Secondly, such diverse mixed-severity disturbance regimes, and the associated dynamics, make old trees and diverse disturbance legacies prevalent in naturally dynamic forest landscapes. Here, we review the research evidence concerning these two fundamental characteristics of forest landscapes in northern Europe, comprising Fennoscandia and the neighbouring northwestern area of European Russia.

### Characteristics of natural disturbance dynamics

Variability in forest dynamics and structure in a given landscape over time results from the interplay between various disturbances and diverse post-disturbance successional pathways. Disturbances vary in form (fire, flooding, wind, insect outbreaks, etc.), size, spatial configuration, frequency (return interval), and severity (Kneeshaw et al. 2011). Importantly, the variation in disturbance severity and thereby the patterns of surviving legacy trees need to be taken into account (Pennanen 2002). Even large fires are clearly not as uniform and stand-replacing as previously assumed (Kneeshaw et al. 2011). Rather, low- to medium-severity fires, such as surface fires, are prevalent in northern Europe (Gromtsev 2002; Lampainen et al. 2004; Shorohova et al. 2009; Kuuluvainen and Aakala 2011), and extensive areas may escape fires for very long periods (Zackrisson et al. 1995; Wallenius et al. 2010).

The commonness of non-stand-replacing disturbances is the result of various interrelated top-down and bottom-up factors that control the role of fire. At large scales, fire dynamics vary with climate (Drobyshev et al. 2014; Rolstad et al. 2017; Aakala et al. 2018). For example, fire-return intervals are exceptionally long in semi-oceanic mountain climates (Carcaillet et al. 2007; Aakala et al. 2009; Wallenius et al. 2010). Natural fire barriers, such as rivers, lakes, and wetlands, are abundant and reduce the importance of forests fires in landscapes (Hellberg et al. 2004; Wallenius et al. 2004). The severity and spread of fires are reduced in certain areas due to small surface fuel loads in nutrient-poor, low-productive soils or because of the limited time for fuel buildup caused by recurrent fires (Schimmel and Granström 1997). At small scales, the pattern of variation in fire-return interval varies along gradients in local factors such as soil moisture and understorey vegetation (Zackrisson 1977; Gromtsev 2002; Wallenius et al. 2004).

While acknowledging the wide range of variation in forest disturbance and successional dynamics, these can be grouped into three broad cyclic types (Angelstam and Kuuluvainen 2004): (1) small-scale tree mortality inducing gap dynamics, (2) partial, low-severity stand-scale disturbances inducing tree age-cohort dynamics, and (3) high-severity, stand-replacing disturbances inducing even-aged dynamics. A systematic review of natural forest studies in Fennoscandia (Kuuluvainen and Aakala 2011) showed that gap dynamics are most commonly reported, followed by cohort dynamics. Overall, ca. 80% of reviewed studies reported various non-stand-replacing disturbance dynamics. The same pattern was found in a review of Russian studies (Shorohova et al. 2009).

Gap dynamics are most common at sites with relatively moist and stable microclimates where fires rarely occur (Hörnberg et al. 1995, 1997), but such small-scale dynamics also shape forests on varying site types and tree-species compositions (Wallenius et al. 2004; Kuuluvainen and Aakala 2011). Historically, cohort dynamics were promoted by common low-severity fires and partial tree mortality in dry nutrient-poor Scots pine (*Pinus sylvestris* L.) sites (Zackrisson 1977). Multiaged Scots pine-dominated old forests shaped by frequent, low-severity fires were dominant in middle boreal Swedish landscapes prior to the nineteenth century, after which fire suppression and the expansion of forestry began (Östlund et al. 1997; Linder and Östlund 1998; Axelsson and Östlund 2001). Further, studies in existing, unmanaged forests describe fire-driven cohort dynamics in both middle boreal (Kuuluvainen et al. 2002; Lampainen et al. 2004; Sandström et al. 2020) and northern boreal (Aakala 2018) Scots pine-dominated forest landscapes. However, cohort dynamics may also be found in mesic, intermediate sites, where Scots pine may mix with deciduous trees and Norway spruces (*Picea abies* L.) during succession (Kuuluvainen et al. 2002).

Even-aged dynamics due to severe fires or storms have only infrequently been documented in northern European boreal forests (Niklasson and Granström 2000). Kuuluvainen and Aakala (2011) show that such dynamics are relatively rarely reported, comprising ca. 20% of all reviewed studies. Still, extensive, severe disturbances do occur and create specific diverse, open habitats with very large dead wood quantities (Uotila et al. 2001; Ylisirniö et al. 2012). Such severe disturbances may have an impact on forest structure that last for centuries (Lilja et al. 2006; Aakala et al. 2009).

To conclude, the current evidence shows that variable non-stand-replacing disturbances are the dominant drivers of forest dynamics and NRV in northern European boreal forests. Forest dynamics are characterized by long return intervals of severe disturbances and prevalence of small-scale gap dynamics as well as the variable- or mixed-severity disturbances inducing partial tree mortalities and age-cohort dynamics. The share of non-stand-replacing disturbance dynamics is expected to be large, approximating 2/3 or more at larger scales. Such disturbance regimes and successional dynamics result in complex forest and landscape structures with prevalence of old forests (see next section; Pennanen 2002).

### The prevalence of old forest age classes

The variation in forest age at large scales largely determines the diversity of habitats, species, and ecosystem processes. Here, forest age is determined as minimum time elapsed from last major disturbance (often indicated by the age of the dominant tree cohort). Depending on the disturbance regime, forest age-class distributions vary in space and time, a property that has been conceptualized as their NRV (Landres et al. 1999). However, it should be emphasized that defining forest age in naturally dynamic forests is not straightforward. Most old forests are uneven-aged, composed of not only old trees, but many more younger trees contributing to variable and often multilayered canopy structures. Further, old-growth conditions and legacy structures, including diverse living tree and dead wood structures, develop over time periods much longer than indicated by the age of the oldest trees alone (Lilja et al. 2006; Ylisirniö et al. 2012; Johnstone et al. 2016).

In northern European forests under intrinsic dynamics, large-scale forest age-class distribution patterns may be inferred from various types of studies. Studies based on historical documentation and maps of middle boreal Swedish landscapes show that old, multilayered forests with high densities of large-diameter living and standing dead trees were dominant, comprising ca. 70–95% of the area in the nineteenth century (Östlund et al. 1997; Linder and Östlund 1998; Axelsson and Östlund 2001). Similar conditions prevailed in low human impact areas in middle and northern boreal Finland (Ilvessalo 1927, 1937; Keto-Tokoi and Kuuluvainen 2014; Anonymous 2019). In Finland, timber trees in the mid-nineteenth century had to have a minimum age of 140 years (Keto-Tokoi 2014), suggesting that old trees were a common feature.

The information derived from historical documentation is supported by recent field studies in existing natural reference landscapes. These studies show that Scots pine forests with abundant old trees and multiple tree age cohorts dominate in both northern (Engelmark et al. 1994; Aakala 2018) and middle (Kuuluvainen et al. 2002; Wallenius et al. 2004) boreal reference landscapes shaped by historical fires. Past fires have maintained a spatially and temporally continuous presence of old fire-tolerant Scots pine forests, where trees over 250 years of age are common (Engelmark et al. 1994; Kuuluvainen et al. 2002; Wallenius et al. 2004). Old age classes also dominate Norway spruce forests in both northern (Engelmark et al. 1994; Wallenius et al. 2005; Aakala et al. 2009) and middle (Wallenius 2002; Wallenius et al. 2004) boreal reference landscapes, where fires have been rare or absent. More than 80% of the Norway spruce forests sampled in these studies are typically ≥ 150 years and ca. 50% are ≥ 250 years old, although Norway spruce trees become senescent at 300 years of age (Engelmark et al. 1994; Wallenius 2002).

Available computer simulations support the empirical observations. Pennanen’s (2002) spatially explicit simulation-based analysis of a typical middle boreal landscape predicts that old (≥ 150 years) forests will cover over 50% of forest area under mixed-severity fire disturbance dynamics. This is the case over a range of average fire-return intervals simulated: 240, 150, and 50 years, but so that high fire frequencies favour Scots pine over Norway spruce, while low frequencies favour Norway spruce over Scots pine (Pennanen 2002). Forest landscapes driven by small-scale and/or partial disturbances thus show considerable inertia, resulting in relatively stable NRVs of age-class distributions over time (Pennanen 2002).

To conclude, the accumulated evidence indicates that old forests (uneven-aged with trees at least 150 years old or more) are a prevalent or even dominant feature in naturally dynamic boreal forests in northern Europe. The NRV in the proportion of old forest appears to vary between 50% and 95% when considering the research studies reviewed, with a conservative, low estimate around 50%.

## The ASIO model: An influential standard for naturally dynamic forests

The ASIO model was formulated in the 1990s as an educational tool for managers to explain how natural fire dynamics affect the structure of boreal forests and how this knowledge may be used in managing the forests (Rülcker et al. 1994). The ASIO model is attractive because it implies setting stand-level, bottom-up targets rather than landscape-level, top-down targets for management. The basic assumption is that site type is the main determinant of natural fire dynamics (Angelstam 1998). Thus, the model may easily be implemented at the stand level and scaled up to landscape or regional levels using standard forest inventory data on forest stand site types.

### A guide for management

**Fig. 1:**
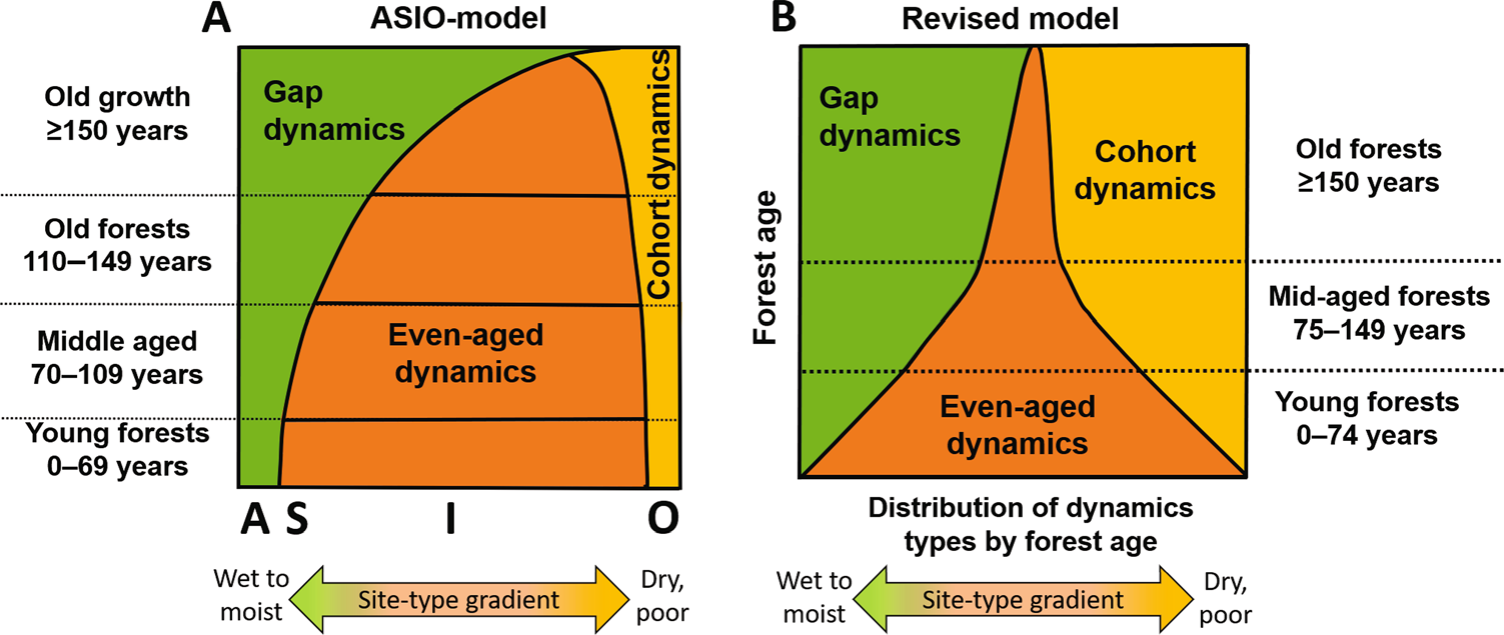
Illustration of the properties of and the differences between the ASIO model and the revised reference model. **A** The ASIO model by Angelstam (1998), demonstrating how the areal proportions of forest dynamics types are distributed in forest age classes (*y*-axis) and site type-related ASIO classes (*x*-axis). This model emphasizes the dominant role of stand-replacing disturbances and even-aged dynamics (orange areas) and the prevalence of forests younger than 150 years. **B** The revised reference model, reflecting current understanding of distribution of forest dynamics types by age classes, emphasizes the greater importance of non-stand-replacing disturbances, gap dynamics (green areas), and cohort dynamics (yellow areas). In the revised reference model (**B**), the dynamics types are less strictly related to site type and there is a larger share of old and old-growth forests when compared with the ASIO model formulation (**A**). The cover of coloured areas reflects the landscape-level share of area (%) of the three broad types of disturbance dynamics and the three major forest age classes given by the revised reference model in Table 1

The acronym ASIO refers to four classes of fire occurrence frequency: Absent, Seldom, Infrequent, and Often (Rülcker et al. 1994; Angelstam 1998; Fig. 1). At one end of the site-type gradient (Arnborg 1990), class A occurs mainly on wet to moist site types, where *Sphagnum* spp. mosses typify the ground vegetation. Class A is considered “non-fire refugia”, with fire intervals > 300 years, where uneven-aged, late-successional forests governed by gap dynamics are assumed to prevail. Class S also occurs on moist but more upland site types, where stand-replacing fires induce even-aged dynamics during extreme droughts at intervals of approximately 200 years. On the opposite end of the gradient, class O occurs on dry, poor site types, characterized by *Cladonia* spp. lichens as ground vegetation. Class O is assumed to be affected by low-severity fires with intervals of 40–60 years. Scots pine-dominated forests shaped by cohort dynamics are expected to be typical of this class. Class I occurs on all other site types between these two extremes. It includes common mesic, intermediate to rich site types with variable ground vegetation, i.e. various pleurocarpus mosses and mixtures of dwarf shrubs, graminoids, or herbs. Even-aged forests formed by stand-replacing fires with intervals just below 100 years are considered characteristic. Hence, one type of disturbance dynamics of a certain frequency and severity is assumed to prevail on the site types of each fire frequency class. In essence, the ASIO model assumes a dominance of stand-replacing disturbances and even-aged dynamics on mesic, intermediate to rich site types, which comprise the bulk of forestland area (Angelstam and Kuuluvainen 2004).

The ASIO model has been used to guide managers on how to stratify forestry practices based on site types to emulate natural boreal forest conditions (Fries et al. 1997). Old forest conservation (reserves) or selection logging are proposed for imitating gap dynamics within class A (Rülcker et al. 1994; Angelstam 1998). However, even-aged forest management with low-retention clear-cutting is recommended as the main management method for all other classes. Clear-cutting with shelter wood systems is suggested for class S and clear-cutting with seed-tree retention for class O. Importantly, conventional clear-cutting with forest rotations around 100 years is proposed as the main cutting method for class I.

When comparing the current understanding of forest reference conditions to the assumptions of the ASIO model, a clear mismatch may be seen. The ASIO model formulation overemphasizes the role of even-aged forest dynamics and underestimates the role of partial and small-scale disturbances (Fig. 1). A major deviance compared to current understanding is the assumption that even-aged dynamics dominate on the main part of the forestland area, consisting of mesic, intermediate to rich site types.

### A bottom-up logic for estimating reference conditions and conservation area needs

The bottom-up logic underlying the ASIO model has been used for estimating reference conditions and forests reserve needs in Sweden (Angelstam and Andersson 2001, with details explained in SOU 1997:97 and 1997:98) and Estonia (Lõhmus et al. 2004). The assumed natural occurrence of forests developing from different types of disturbance regimes is first determined from data on site type distribution. The distribution of forest developmental stages after disturbance in terms of age classes is then estimated by using models of equilibrium forest dynamics. Finally, the regional reserve need is computed as 20% of the estimated area of those forest types and developmental stages which are considered “management incompatible”, i.e. not possible to maintain with conventional even-aged low-retention forestry. The threshold value of 20% reflects expected minimum habitat levels needed to ensure sufficiently connected functional habitat networks and viable populations of specialized species that cannot persist in managed forests. Management incompatible forests are considered to be those older than the age stipulated for final felling (the rotation age), i.e. 110 years in the Swedish analysis. By contrast, younger forests are assumed to be management compatible and maintain their key ecological characteristics under low-retention forestry (Angelstam and Andersson 2001).

**Fig. 2:**
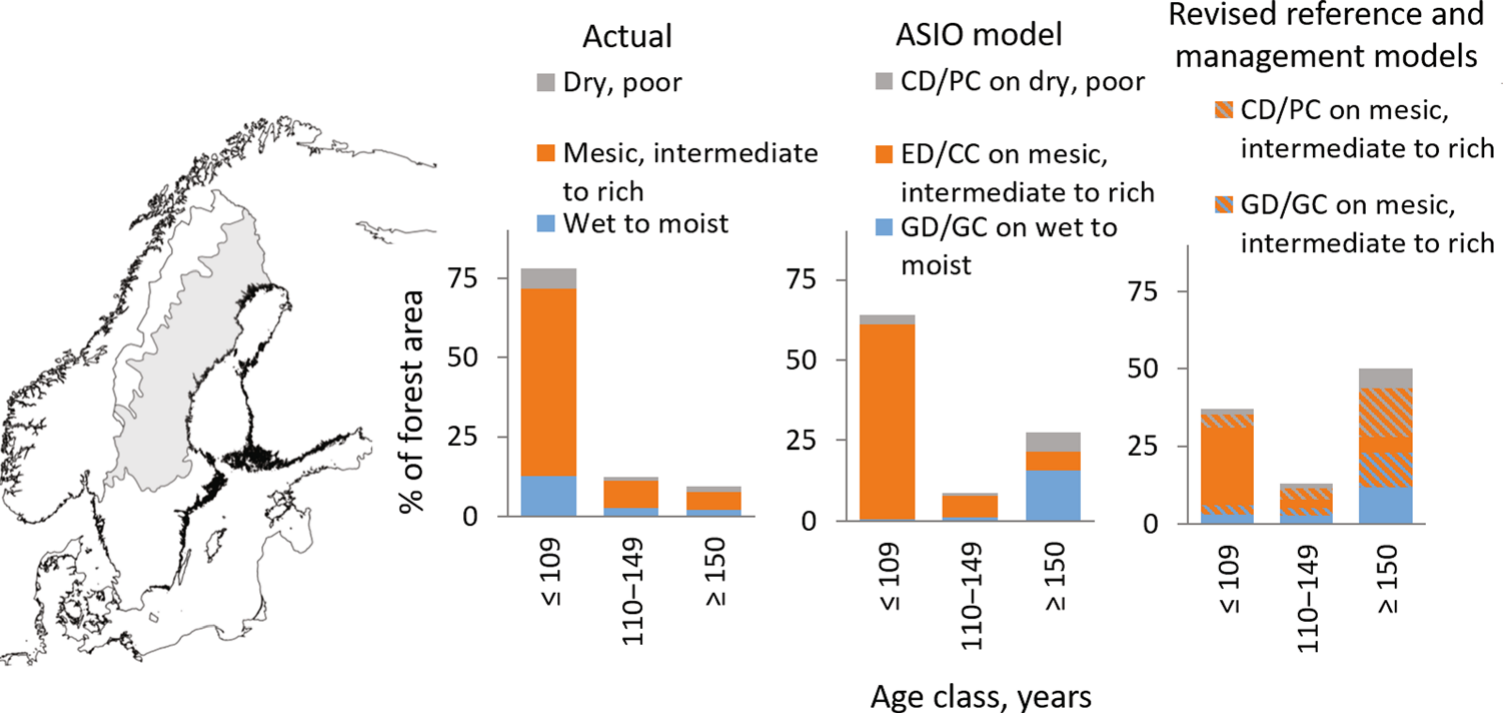
Illustration of the age-class distribution of mineral soil forests in the northern part of the boreal region in Sweden (the grey area of the map). The left diagram shows the actual age-class distribution across three major site types. The middle diagram shows the distribution estimated using the stand-level and bottom-up logic underlying the ASIO model, i.e. assuming that a single type of disturbance dynamics; gap dynamics (GD), even-aged dynamics (ED), or cohort dynamics (CD), prevails on each major site type. Further, for even-aged dynamics on the dominating part (mesic, intermediate to rich site types), the age-class distribution is based on averaging the negative exponential and Weibull distributions of stand-replacing disturbances with a return interval of 100 years (cf. Angelstam and Andersson 2001; Angelstam and Kuuluvainen 2004). As a consequence, a dominance of young (0–109 years) forests is expected. The use of forest management with gap cutting (GC), clear-cutting (CC), and partial cutting (PC) to emulate the dynamics are indicated. The right diagram shows the results when using the revised reference model (Table 1 and Fig. 3). It emphasizes a prevalence of old forests (≥ 150 years) due to a greater importance of non-stand-replacing disturbances and gap and cohort dynamics. Further, the three dynamics types are less strictly related to site type. The corresponding management model prescribes that their targeted distributions (1/3 of each dynamics type) can be attained by allocating the three cutting methods within each age class by taking the available site-type distribution and their probable natural dynamics into account (e.g. gap dynamics is more common on moist to wet site types, etc.). Still, gap and cohort dynamics need to be emulated not only on moist to wet and dry, poor site types but also on mesic, intermediate to rich site types (hatched areas). See Appendix S2 for details on how age-class distributions were derived

We exemplify outcomes of the bottom-up logic with a case study of boreal forests on mineral soils in the northern part of Sweden (see Appendix S1-2 for details). A large part of the region’s forests is in a transition phase due to forestry, but particularly the remote inland zone still hosts large areas of natural or near-natural forests (Svensson et al. 2019). However, NFI estimates of the age-class distribution indicate that the region is dominated by young (0–109 years; 78%) and mid-aged (110–149 years; 13%) forests, while old forests (≥ 150 years, 9%) are scarce (Swedish NFI 2020; Fig. 2). Such a clear prevalence of young and mid-aged forests is an expected effect of governance by modern forestry with clear-cut rotations of ca. 100 years (Kuuluvainen et al. 2015).

We use the rationale of the previous Swedish analysis (Angelstam and Andersson 2001), where three broad types of disturbance dynamics are assumed to prevail on three major site types: gap dynamics on wet, cohort dynamics on dry and even-aged dynamics on mesic site types (cf. Angelstam and Kuuluvainen 2004). Mesic, intermediate to rich site types cover ca. 73% while moist to wet and dry, poor sites types cover ca. 27%. Thus, the bulk of forest is assumed to be mainly shaped by even-aged dynamics and given an age-class distribution estimated as an average between the negative exponential and Weibull distributions resulting from stand-replacing disturbances with a return interval of 100 years. As a consequence, a dominance of young (64%) forests is predicted. Still, the share of so-called management incompatible forests ≥ 110 years (36%) is expected to about 1.5 times larger than it actually is in the region (22%; Fig. 2). This is mainly explained by the relatively large proportion (27%) of wet to moist and dry, poor site types, where non-stand-replacing dynamics and forests ≥ 110 years are predicted to prevail under natural conditions. The estimated reserve need is then ca. 7% when considering forests ≥ 110 years only (20% of 36%), but nearly 8% if we include also younger forests shaped by gap or cohort dynamics (20% of 40%) as management incompatible (see Appendix S1-2). Similarly, Angelstam and Andersson (2001) predicted a reserve need at 8–9% when restricting their analysis to forests with a timber production ≥ 1 m^3^/ha/year.

## Revised models based on current understanding

In the ASIO model, following the bottom-up logic, the occurrence of natural forest dynamics types is largely determined by site type at the stand level (Fig. 1). The landscape-level distribution of forest dynamics types is simply a result of bottom-up summing from the stand level. However, this is a gross simplification of reality, as the disturbance regime is fundamentally a large-scale phenomenon, including chance events and complex cross-scale spatial interactions (Bergeron and Fenton 2012; Burton 2013). For estimating ecologically realistic reference conditions for landscapes, the bottom-up approach represented by the ASIO model therefore needs reconciliation with a large-scale, top-down perspective.

Below we present a revised model for boreal forest reference conditions in northern Europe, based on current understanding of natural forest dynamics and structure. The revised reference model is then translated into a management model aiming to maintain representative types of boreal forests. We also show how the revised reference model can be used as a basis for estimating conservation area needs. Critically important natural dynamics properties of boreal forests, such as variability in disturbance frequency and severity, and resulting structures, are incorporated in the reference model, but in a simplified form to warrant practical applications. Because forests always exhibit natural variation, the model must not be considered a rigid target, but rather a reference toward which the forest should converge.

### The reference model

**Fig. 3:**
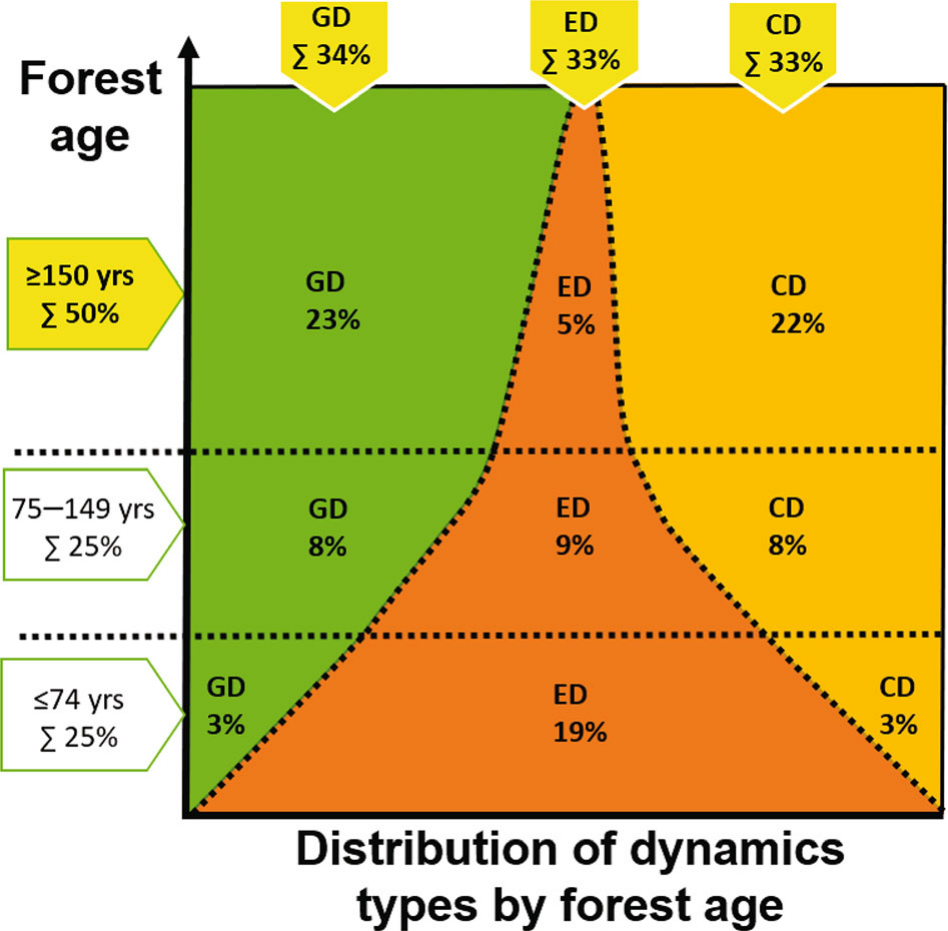
Illustration of the revised reference model, showing the landscape-level distribution (% of area) of the forest age classes (*y*-axis) as a function of disturbance dynamics in terms of three broad dynamics types (Green GD: gap dynamics; Orange ED: even-aged dynamics, Yellow CD: cohort dynamics) (*x*-axis). The cover of coloured areas reflects the share (% of area) of three broad types of disturbance dynamics and three major forest age classes given by the revised reference model in Table 1. The dominance (at least 50%) of old forests (≥ 150 years), but also the equal shares (1/3 or 33.3%) of the three disturbance dynamics types comprise basic model settings and overall targets (highlighted figures in bold) derived from a review of current understanding of reference conditions (see text, Table 1 and Appendix S1 for further explanation). The model is not considered an absolute target but rather a general reference toward which management should aim

The revised reference model is based on our review of natural forest dynamics and structure. It is defined by two fundamental components: (1) the distribution of forest age classes in a given landscape or region and (2) the distribution of forest dynamics types between and within forest age classes (Fig. 3; Table 1). The first component introduces a top-down target for the age-class distribution. The second component aims at incorporating the bottom-up processes of complexity in forest dynamics, including the prevalence of gap and cohort dynamics due to non-stand-replacing disturbances. Together they ensure the maintenance of the desired level and variability of key features of natural forest dynamics and structure at both stand and landscape levels.

**Table 1:**
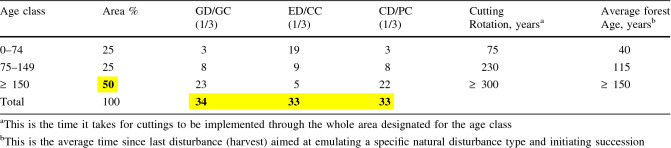
The key properties of the revised reference model and the corresponding management model viewed across large scales. The reference model includes the distribution (% of area) of three major age classes and three broad types of forest disturbance dynamics (GD: gap dynamics; ED: even-aged dynamics; CD: cohort dynamics). The dominance (at least 50%) of old forests (≥ 150 years), but also the equal shares (1/3 or 33.3%) of the three disturbance dynamics types comprise basic model settings and overall targets (highlighted figures in bold) derived from a review of current understanding of reference conditions. The three disturbance dynamic types are then distributed across age classes based on the principle that non-stand-replacing disturbance dynamics prevail in old forests while even-aged dynamics dominate in young forests. The main part (2/3) of gap and cohort dynamics occurs in old forests (2/3 of 1/3 or 22.2% of each type) and their remaining shares (11.1%) are distributed across young (0–74 years; 2.8%) and mid-aged (75–149 years; 8.3%) forests so that they increase linearly with increasing age. Even-aged dynamics are distributed in the opposite way (19.4%, 8.3% and 5.6% in young, mid-aged and old forests, respectively). Rounding to even percentages is done according to the same principle (see Appendix S1 for details). Here, young and mid-aged forests are separated at 75 years, but any other age limit may be used while the share of disturbance dynamic types change linearly until forests become old. The use of the corresponding cutting methods to emulate the reference forest dynamics and structure are indicated (in red; GC: gap and selection cutting; CC: clear-cutting; PC: partial cutting, i.e. removal of a portion of the tree volume so that an uneven-aged stand of trees remain) along with the approximate rotation time and target average age for each age class under management. The revised reference model is not considered a strict target but rather a reference toward which the managed forest should converge over time

**Table 2:** Application steps of the proposed management model for maintaining and/or restoring natural-like structures of northern European boreal forests at the landscape or regional scale. The application may be clarified and divided into steps that form an adaptive management cycle (in the face of uncertainty)

Delineate the landscape or region for management (this may be whole landscapes or a particular targeted proportion of a region) Allocate the areas for the three major forest age classes (according to Table 1) Allocate the three cutting methods (gap cutting, clear-cutting, and partial cutting, each covering approximately one-third of the delineated landscape) within each age class, to emulate the mix of dynamics, taking the available site-type distribution and their probable natural dynamics into account (e.g. gap dynamics more common on moist to wet site types, etc.) Determine cutting rotations to attain desired age structures in areas designated for each of the (nine) forest age-class/forest dynamics type—combinations (Table 1) Apply management through time Monitor and modify management if needed	

#### Forest age-class distribution

To warrant practical application, the age-class distribution of the revised reference model must be realistic, quantitative, and relatively simple. Hence, the reference conditions are modelled using three major forest age classes: young 0–74, mid-aged 75–149, and old ≥ 150 years (Fig. 3; Table 1). The area proportions across age classes adhere to current understanding of natural forest age distribution at the large scale, i.e. with a representative distribution of different types of forests and disturbance dynamics. The critically important difference compared to previous models is the large overall share (at least 50%) of old forest (Figs. 1 and 3). The remaining part (50%) is evenly distributed into young and mid-aged forests, here separated at 75 years, i.e. an age making the classes equally wide but also matching final cutting ages (ca. 80 years) in both northern Finland and Sweden. Note that age of the dominant tree cohort is used as overall descriptor of natural forest developmental patterns and structures. Thus, age is assumed to correlate with natural stand structure complexity, i.e. heterogeneous legacy structures with multiple tree ages, multilayered canopy structure, and a diverse supply of dead wood.

#### Forest dynamics types

The model assumes that forest dynamics fall into three broad types: gap, even-aged, and cohort dynamics (Figs. 1 and 3; Table 1). Site type and forest age are assumed to mainly determine the occurrence of these dynamics types in the landscape. However, non-stand-replacing disturbance dynamics are expected to be prevalent together with mixtures of even-aged dynamics driven by relatively rare, high-severity disturbances. The important model assumption is that all types of forest dynamics may occur on all major site types, although in different proportions. This assumption is in line with the stochastic nature of disturbance dynamics.

The relative importance of the three dynamics types is inferred from a comprehensive review of studies on natural forest dynamics in Fennoscandia (Kuuluvainen and Aakala 2011, and references therein) showing that gap dynamics are most commonly reported (ca. 50% of studies), followed by cohort (30%) and even-aged dynamics (20%). However, the prevalence of even-age dynamics may be underestimated in this analysis, as studies are preferably performed in old forests. To correct for this potential bias, equal proportions (1/3) of each type are used as overall, general targets (Fig. 3; Table 1). Still, the larger share (2/3) of non-stand-replacing disturbances is a critical difference compared to earlier model formulations.

Further, the proportions of the forest dynamics types vary between forest age classes (Fig. 3; Table 1). Non-stand-replacing disturbance dynamics are common in old forests, while even-aged dynamics are dominant in young forests. We assume that even-aged structures are most common in young forests affected by stand-replacing disturbances, but gradually decreases in importance with time during forests ageing due to increasing competition, low-severity disturbances and tree recruitment (Lilja et al. 2006; Aakala et al. 2009). Hence, the main part (2/3 of 1/3 or 22.2%) of gap dynamics along with cohort dynamics occurs in old forests, and the remaining share of each type is distributed so that it increases linearly with increasing age across young (3% of each type) and mid-aged forests (8%). The share of even-aged dynamics is distributed in the opposite way, i.e. it decreases from young (19%) to mid-aged forests (9%) to become insignificant (5%) in old forests. Young and mid-aged forests are separated at 75 years, but any other age limit may be used while the importance of the disturbance dynamic types change linearly until forests become old (see Appendix S1 for details).

### The management model

The basic idea is to apply cutting methods and forest rotations that mimic natural forest dynamics, their ecological impacts, and proportions at large scales. The model considers managing for three forest age classes, each incorporating three types of forest dynamics, yielding a total of nine forest age-class/forest dynamics type –combinations (Tables 1, 2).

#### Choosing harvesting methods

The stand-level management is based on choosing cutting methods to emulate natural forest disturbances that drive specific forest dynamics types. It is important to retain a sufficient amount of natural-like legacy structures of living and dead trees in all harvesting operations (Gustafsson et al. 2012; Johnstone et al. 2016). Gap and partial cutting to emulate small-scale and partial disturbances and clear-cutting with retention to emulate stand-replacing disturbances are each carried out on one-third of the landscape, respectively. Hence, around two-thirds of the landscape is managed with harvesting methods with disturbance severity levels lower than clear-cutting and stand replacement (Table 1). Heterogeneous uneven-aged forest conditions therefore prevail on a major part of the designated area.

#### Allocating harvesting methods in the field

**Fig. 4:**
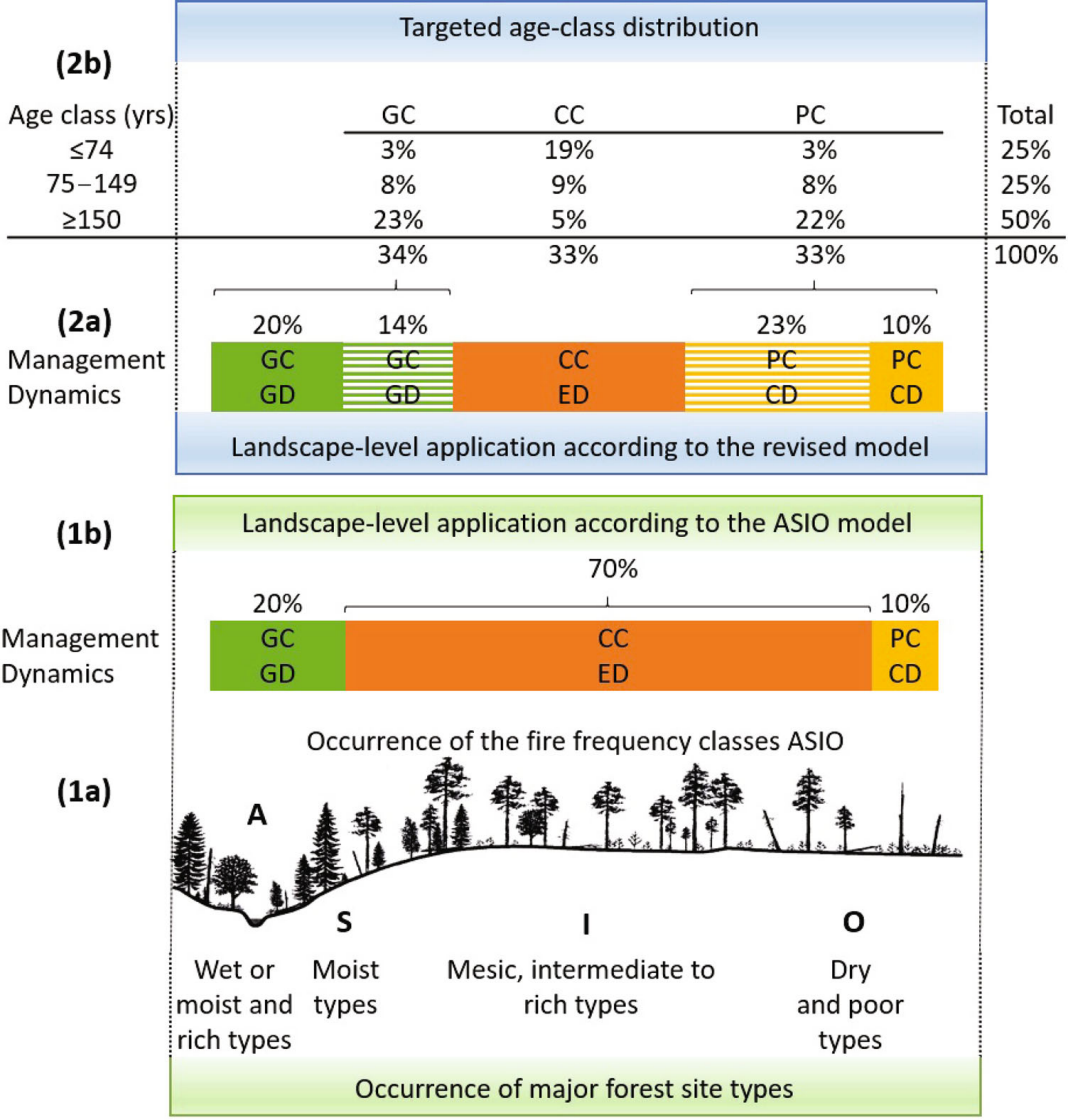
Comparison of the ASIO model formulation (lower panel) and the revised reference model (upper panel). The lower panel (**1a**) shows how the ASIO model (Angelstam 1998) predicts that the three types of forest dynamics (Green GD: gap dynamics; Orange ED: even-aged dynamics, Yellow CD: cohort dynamics) are distributed as a function of site-type gradient. Panel (**1b**) shows a tentative landscape-level distribution (% of area) of the three forest dynamics types and corresponding imitation cutting methods (GC: gap cutting; CC: clear-cutting; PC: partial cutting). The top panel shows the revised reference model based on the current understanding of intrinsic forest dynamics and age-class distribution in northern European conditions. The three dynamics types occur in equal proportions (1/3 of each type) and are less strictly related to site type. The corresponding management model prescribes that cutting methods can be allocated in field by taking the available site-type distribution and their probable natural dynamics into account (e.g. gap dynamics is more common on moist to wet site types etc.). A key difference between the management models is the larger share of non-stand-replacing harvesting on mesic, intermediate to rich site types (GC and PC; hatched areas) in the revised model compared to the ASIO model formulation (Panel (**2a**)). Panel (**2b**) shows the targeted landscape-level age-class distribution to be achieved by varying cutting methods and forest rotations among the three types of management. Drawing: J. Karsisto

To allocate management onto landscapes with varying site-type distributions so that the targeted reference conditions are attained, the landscape is first spatially divided into nine forest age-class/cutting type –combination areas in proportions shown in Table 1. At stand-level, the cutting types are allocated as inspired by the ecological understanding of the natural occurrence of disturbance types along the site-type gradient (Zackrisson 1977; Wallenius et al. 2004); e.g. single-tree or fine-scale gap cuttings on moist to wet site types and partial cuttings on dry, poor site types. However, forests on mesic, intermediate to rich site types covering the bulk of the landscape are managed by mixtures of gap and partial cuttings in addition to clear-cutting (Table 1; Fig. 4).

#### Maintaining desired forest age-class distribution

The targeted age-class distributions in each of the nine age-class/cutting type -combinations are attained by implementing variable, area-based cutting rotations (Table 1). For example, in areas allocated for young forests, all three types of cuttings are implemented so that an approximately 75-year average rotation and 40-year average forest age are achieved for these areas. In young forests designated for gap and partial cuttings, 20–30% of the forest area is harvested approximately in cutting cycles of 20–30 years, which results in a small-scale mosaic of different-aged forest. Young forests designated for clear-cutting will become a coarse scale mosaic. Likewise, in areas allocated for mid-aged and old forests, extended rotations are applied in all three cutting methods, with the aim of maintaining old trees and mosaic structures typical of old-growth forests (Table 1).

### A basis for estimating conservation area needs

For illustration, we continue our case study of boreal forests in northern Sweden (Fig. 2). To highlight important features of the revised reference model, we compare its outcomes with the results we obtained in our previous analysis using the bottom-up logic underlying the ASIO model. To facilitate this, we adapt the revised reference model to the classification of young (0–109 years) and mid-aged (110–149 years) used in our previous analysis (see above and Appendix S1-2 for details).

The revised reference model predicts 13% mid-aged (110–149 years) and 50% old (≥ 150 years) forests, respectively. Thus, under natural disturbance regime, at least 63% of forests are expected to be ≥ 110 years. This is clearly higher than estimated (36%) using the ASIO model. Particularly the shift towards old forests (≥ 150 years) becomes prominent with the revised reference model. Their share (at least 50%) is expected to be roughly twice as large as that estimated (28%) with the ASIO model and nearly six times larger than what actually exists today (9%; Fig. 2). The new estimates of reference conditions also imply a significant reduction in the proportion of young (37%) forests compared to what is expected based on the ASIO model (64%) or actually found (78%). Furthermore, to achieve the model’s estimated prevalence of old forests (at least 50%), gap and cohort dynamics (totally 67%) are much more important than expected (27%) with the ASIO model’s bottom-up logic based on the actual site-type distribution (Fig. 2). Finally, the estimated reserve need defined as 20% management incompatible natural forests is nearly 13% when considering forests ≥ 110 years only (20% of 63%), but 15% when including also younger forests shaped by gap or cohort dynamics (20% of 75%; see Appendix S1-2). This is about twice as high as estimated with the bottom-up logic of the ASIO model.

## Discussion

Realistic models of forest dynamics and structure are vital for defining reference forest conditions for ecosystem management and biodiversity conservation (Angelstam 1998; Pennanen 2002). Such models need to account for key ecological interactions in disturbance and forest succession dynamics across multiple scales, such as the stand, landscape, and regional scales. Recent research in northern Europe (Kuuluvainen and Aakala 2011) and elsewhere in the boreal zone (Kneeshaw et al. 2011; Bergeron and Fenton 2012; Burton 2013) emphasizes the importance of low- and moderate–severity disturbances in shaping forest structural complexity and age distribution at the landscape scale (Kuuluvainen 2009). This observation differs drastically from earlier views, considering stand-replacing disturbances and even-aged forest dynamics as the norm in boreal forests (Sirén 1955; Zackrisson 1977; Angelstam 1998, Mielikäinen and Hynynen 2003).

In northern Europe, the ASIO model and its bottom-up approach has gained the status of a “standard” reference promoting the use of natural forest dynamics for ecosystem management and conservation (Angelstam 1998; Lõhmus et al. 2004). Clearly, the ASIO model represented a novel approach and innovation in forest management. However, its formulation has contributed to the fortitude of the view that boreal forests are intrinsically dominated by even-aged dynamics that occur within delineable compartments of specific site types. Because these assumptions have proven to be flawed in northern European conditions, the bottom-up approach represented by the ASIO model results in estimates of forest reference conditions that fall outside their actual NRV (Kuuluvainen 2009).

### Introducing a top-down perspective on reference conditions

The ASIO model formulation evidently needs to be revised and reconciled with current ecological understanding to estimate realistic reference conditions for boreal forests. To this end, we present a revised reference model, which ensures a top-down perspective by introducing large-scale targets for the prevalence of non-stand-replacing disturbance dynamics (67% of area) and the associated dominance of old forests characteristics (at least 50% of area; Table 1). We accentuate that these overall targets are conservative and may be much higher, for instance, in regions with semi-oceanic mountain climates, as in western Fennoscandia (Angelstam 1998), where fire return intervals are exceptionally long (Carcaillet et al. 2007; Aakala et al. 2009; Wallenius et al. 2010) and extensive areas may escape stand-replacing fires for very long periods (Zackrisson et al. 1995; Wallenius et al. 2010).

One important implication of the top-down perspective is that the occurrence of different forest dynamics types are less strictly related to site type than in the ASIO model, where their landscape-level distribution is a result of simple bottom-up summing from the stand level. Thus, the top-down perspective on reference conditions remains decisive even if the site-type distribution changes due to forest management or other human activities. In fact, such changes have taken place in northern Sweden where the area of reindeer lichen-rich, poor site types have declined by 75% during the past 50 years due to effective fire suppression (Sandström et al. 2016). Likewise, the area of swamp forests, i.e. forests on wet to moist sites types, has successively declined since the 1990s (Kempe and Dahlgren 2016). Hence, under such changes, models solely relying on a bottom-up summing from current site-type distribution will result in biased estimates of reference conditions.

### Highlighting the need of adaptive management for old forest characteristics

The current systematic use of clear-cutting and short rotation, even-aged management systems in northern Europe and elsewhere has been criticized due to its negative effects on forest structure and ecological processes (Kuuluvainen et al. 2012) as well as biodiversity (Angelstam et al. 2020). Clearly, changes to management are necessary to move landscapes toward their expected NRV and thereby improve the provision of forest ecosystems and the services they provide (Peura et al. 2018).

Our review shows that more forests need to be managed with less intense harvesting methods and longer forest rotations than generally used today (Fig. 2). Based on our revised reference model, we outline an ecosystem management model for maintaining a desired large-scale habitat and forest age-class distribution by emulating natural ecosystem dynamics using variable management methods and harvesting techniques (Table 1). In essence, it prescribes that extensive areas are managed for presence of old trees and heterogeneous mosaic structures typical of late-successional old-growth forests.

The top-down perspective introduced by the model implies that there is an increased need of adaptive landscape planning. The management of individual stands cannot only consider local site factors. It must be done with regard the overall targets set at the landscape level (Table 1) and the reference conditions toward which the forest should converge. Furthermore, it is noteworthy that the management model relies on the assumption that forest dynamics is mainly driven by nature-emulation harvest disturbances, and natural disturbances do not play a significant role, as is the case in intensively managed forests of northern Europe. However, when natural disturbance occurs, the management plan must be adjusted, depending on type and severity of disturbance. For instance, large-scale disturbances, such as megafires and severe bark beetle outbreaks, have recently occurred in Fennoscandia (Kärvemo and Schroeder 2007; Gustafsson et al. 2019), and such events are anticipated to become more common with the warming climate (Kuuluvainen and Gauthier 2018). Such disturbance events may still be considered to be part of long-term NRV and they may be incorporated in the management model by reducing the need of managing for similar forest age-class/dynamics type -combinations (Table 1).

### Addressing the representation of biodiversity conservation areas

We emphasize that the main contribution of the revised reference model is that it addresses the regional representation of natural variation necessary for ecosystems and their associated biodiversity to persist over time. Hence, the model is semi-quantitative as it targets an expected representative distribution of different forest dynamics types and developmental stages (i.e. forest age-class/forest dynamics type –combinations) within NRV.

Ignoring the need for representativeness may lead to biased estimates of how much and what types of forests need to be set aside from management to ensure sufficiently connected functional habitat networks for specialized species (Hanski 2011; Angelstam et al. 2020). We therefore recommend that the revised reference model is used in future strategic analyses of regional representation of biodiversity conservation areas in northern Europe. We demonstrate how the model can be applied by using it for estimating the need of forest reserves with the same methodology as in previous analyses (Angelstam and Andersson 2001; Lõhmus et al. 2004). Our results indicate that the reserve need is about twice as large as previously estimated. Clearly, previous analyses have severely underestimated the need of reserves solely due to the fact that the underlying models of forest dynamics underestimate the role of non-stand-replacing disturbances and their effects on forest age structure.

In fact, we argue that the revised reference model should serve as a basis for analyses of needs of regional reference areas for favourable conservation status of boreal forest habitat types under the EU Habitats Directive, which is central to the EU’s biodiversity strategy for implementation of the Convention on Biological Diversity. The revised reference model can be used to estimate area needs of habitat types that both maintain a representative large-scale habitat mosaic (the coarse filter) (Kuuluvainen 2009) and cover a proportion of the landscape needed to ensure habitat network functionality (Angelstam et al. 2020).

## Conclusions

Our review concerning the current understanding of reference conditions of boreal forests in northern Europe reveals a striking change from earlier perceptions emphasizing the dominance of even-aged forest dynamics driven by stand-replacing disturbances towards a view highlighting the variability of disturbance types and severities, and forest successional pathways. Especially, the prevalence of variable non-stand-replacing disturbance dynamics is highlighted. Such diverse dynamics maintain old forest characteristics (with trees aged at least 150 years) as a key component of naturally dynamic northern European forest landscapes.

The novel understanding of boreal forest dynamics and structure has far-reaching consequences for sustainable forest management, landscape restoration, and conservation planning. We present a revised model for defining forest reference conditions. We use this reference model to outline a management model for emulating naturally dynamic forest conditions at landscape level, but also as a basis for estimating the regional needs of representative conservation areas such as reserves. We conclude that attaining sustainable forest management and favourable conservation status in the boreal forests of northern Europe calls for increasing emphasis on management based on intermediate disturbance severity levels and conservation of old, naturally dynamic forests.

## Electronic supplementary material

Below is the link to the electronic supplementary material.

Supplementary material 1 (PDF 934 kb)
