# Supplementary material for: Representative boreal forest habitats in northern Europe, and a revised model for ecosystem management and biodiversity conservation
Source: Ambio. 2021 Jan 17;50(5):1003–17. doi: 10.1007/s13280-020-01444-3 (PMC8035375; doi:10.1007/s13280-020-01444-3)
Supplement: Supplementary file 1 — Supplementary material 1 (PDF 934 kb) [file 13280_2020_1444_MOESM1_ESM.pdf]

Ambio

Electronic Supplementary Material

This supplementary material has not been peer reviewed.

Title: Representative boreal forest habitats in northern Europe, and a revised model for ecosystem management and biodiversity conservation

Appendix S1: Additional explanations of the basics of the revised reference model

The revised reference model is defined by two fundamental components: (1) the large-scale distribution of forest age classes and (2) the distribution of forest dynamics types between and within forest age classes. Two of the overall model targets are based on our review in sections “The prevalence of old forest age classes” and “Characteristics of natural disturbance dynamics” of the article. The targets are, first, at least half of the forests (1/2 or 50%) is old (≥150 years). Second, each of the three disturbance dynamics types (gap dynamics, cohort dynamics and even-aged dynamics) impact one-third (1/3 or 33.3%) of the forest land area. These overall targets form the key settings of the basic model (**Fig. S1**). We further assume that younger forests (<150 years; 1/2 or 50%) are evenly distributed across age and divide them into two equally wide age classes separated at 75 years, young (0–74) and mid-aged (75–149 years) forests (1/4 or 25% or each age class).

Addressing the distribution of disturbance dynamics types across age classes is based on the principle that even-aged dynamics and forest structures are considered most common in post-stand-replacing disturbance sites, but diminish toward older forests due to competition, small-scale and partial disturbances and continuous seedling recruitment (Lilja et al. 2006; Aakala et al. 2009; Ylisirniö et al. 2012). Gap and cohort dynamics as well as uneven-aged structures will for the same reasons increase with time since last major disturbance. We therefore assign the main part (2/3 of 1/3 or 22.2%) of gap dynamics as well as cohort dynamics to old forests (**Fig. S1**). The remaining part (1/3 of 1/3 or 11.1%) of each type is distributed so that it increases linearly with increasing age from young (1/36 or 2.8% of each type) to mid-aged forests (3/36 or 8.3%). While the remaining part is 11.1%, the proportion must increase from 0% to 0.15%, i.e. by nearly 0.001% per year, across the 150 year time span covered by these two age classes (0.15% over 150 years). The proportion at 75 years, i.e. the age limit between young and mid-aged forests, is therefore ca. 0.07% (0.001% per year×75 years). Hence, the cumulative proportion must be 2.8% (0.07%×75 years/2; corresponding to the area of a right triangle) in young forests and 8.3% in mid-aged forests (11.1%-2.8%). To get the proportions of even-aged dynamics, we subtract the proportions of gap dynamics and cohort dynamics from the total proportion assigned for each age class concerned (e.g. 25%-2.8%-2.8% = 19.4% in young forests). The proportions of even-aged dynamics are 19.4%, 8.3% and 5.6% in young, mid-aged and old forests, respectively.

The exact proportions calculated are finally rounded to even percentages. This is done so that summed proportions across dynamic types and age classes equal the overall targets, but also with regards to the principle that non-stand-replacing disturbance dynamics prevail in old forests while even-aged dynamics dominate in young forests (**Fig. S1**).

Here, young and mid-aged forests are separated at 75 years, but any other age limit may be used while the share of disturbance dynamic types change linearly until forests become old. For instance, if we change the age limit to 110 years, the proportion of gap dynamics as well as cohort dynamics at that age will be ca. 0.11% (0.001% per year×110 years; see above). Hence, the cumulative proportion of each dynamics type will be ca. 6% (0.11%×110 years/2) in young forests and in 5% in mid-aged forests (11.1%-6%). Finally, while young forests (0–109 years) will cover 36.7% (110/150 or 73% of 50%) and mid-aged forests (110–149 years) will cover 13.3% (40/150 or 26.7% of 50%) of the land area, the proportions of even-aged dynamics will become 24.7%, 3.1% and 5.6% in young, mid-aged and old forests, respectively.

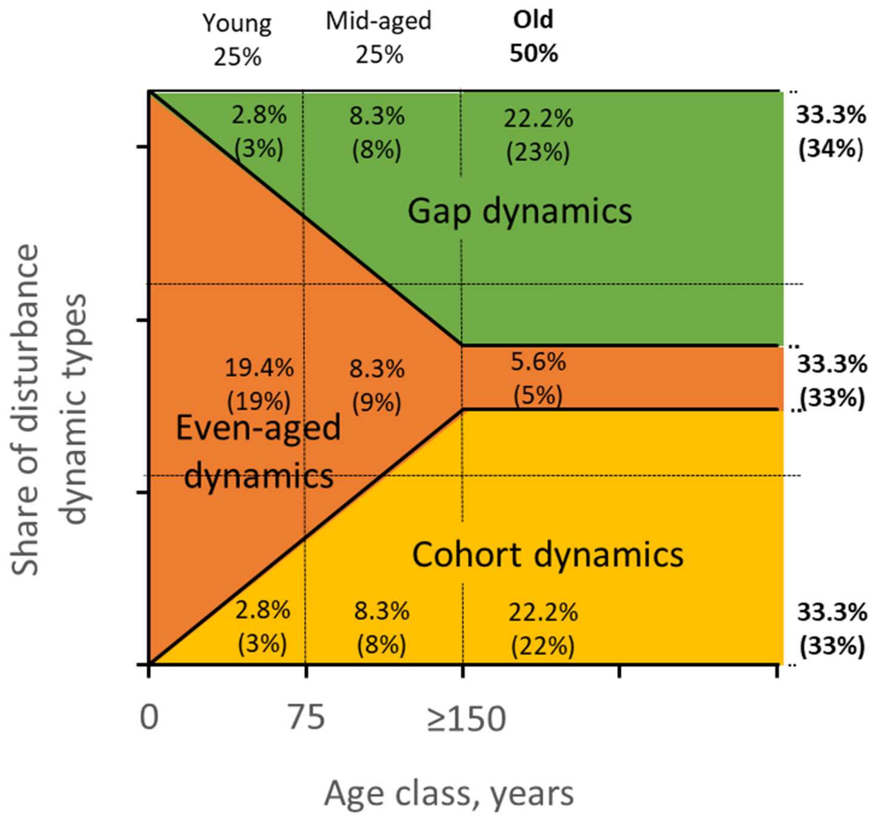

Fig. S1. A schematic description of the revised reference model. The dominance (at least 50%) of old forests (≥150 years), but also the equal shares (1/3 or 33.3%) of the three disturbance dynamics types comprise the basic model settings and overall targets (figures in bold) derived from a review of current understanding of reference conditions. The three disturbance dynamic types are then distributed across age classes based on the ecological principle that gap dynamics and cohort dynamics prevail in old forests while even-aged dynamics dominate in young forests. Their respective proportions change linearly with increasing age across young and mid-aged forests. Here, young and mid-aged forests are separated at 75 years, but any other age limit may be used while the share of disturbance dynamic types change linearly until forests become old. The calculated, exact proportions (figures without parenthesis) are finally rounded to even percentages used in the revised reference model presented in the article (figures in parenthesis). This is done so that summed proportions across dynamic types and age classes equal the overall targets, but also with regards to the principle that gap dynamics and cohort dynamics prevail in old forests while even-aged dynamics dominate in young forests.

Appendix S2: Additional explanation of Figure 2 in the article

The left diagram of **Fig. 2** in the article shows the actual age-class distribution across three major site types of mineral soil forests in the northern part of the boreal region in Sweden (the grey area of the map). The underlying statistics are given in **Table S1**.

Table S1. Shares (%) of three major site types on forest land on mineral soils within the boreal region studied (figures used for the left diagram in **Fig. 2** in the article; data from the Swedish National Forest Inventory 2020). In this example, wet to moist site types include also flooded forests.

Major site types	Total	Young (0–109 years)	Mid (110-149 years)	Old (≥150 years)
Wet to moist types	18	13	3	2
Dry, poor site types	9	6	1.5	1.5
All other (mesic, intermediate to rich) site types	73	59	8	6

The middle diagram of **Fig. 2** in the article shows the age-class distribution estimated using the stand-level and bottom-up logic underlying the ASIO model, i.e. assuming that a single specific type of disturbance dynamics; gap dynamics, even-aged dynamics, or cohort dynamics, prevails on each major site type. The expected age-class distribution under natural conditions in the region studied is computed by multiplying the estimated proportions of each major site type (18%, 9% and 73%; **Table S1**) with three site-type specific age-class distributions (**Table S2**). The results are shown in **Table S3**.

Table S2. The stand-level and bottom-up ASIO logic underlying previous analyses of reference conditions of naturally dynamic boreal landscapes (Angelstam and Kuuluvainen 2004) and long-term forest reserve needs (Angelstam and Andersson 2001, with details explained in SOU 1997:97 and 1997:98) in Sweden. The natural occurrence of forest dynamics types are first linked to specific site types. The expected distribution of forest developmental stages after disturbance in terms of age classes is then estimated by using models of equilibrium dynamics. The age-class distributions of gap and cohort dynamics on wet to moist and dry, poor site types are modelled based on expert judgement (cf. Angelstam and Kuuluvainen 2004). The age-class distribution of even-aged dynamics on mesic site types is modelled as an average between a negative exponential and a Weibull distribution (cf. Johnson 1992) resulting from stand-replacing disturbances with a return interval of 100 years.

Major site type	Disturbance dynamics	Young (0–109 years)	Mid (110-149 years)	Old (≥150 years)
Wet to moist	Gap dynamics, GD	3	7	90
Dry, poor	Cohort dynamics, CD	30	10	60
Mesic, intermediate to rich	Even-aged dynamics, ED	83	9	8

Table S3. The age-class distribution estimated using the stand-level and bottom-up logic underlying the ASIO model (used for the middle diagram in **Fig. 2** in the article), i.e. by multiplying the estimated proportions of each major site type (18%, 9% and 73%; **Table S1**) with three site-type specific age-class distributions (**Table S2**).

Major site types	Young (0–109 years)	Mid (110-149 years)	Old (≥150 years)
Wet to moist	0.5% (18%×3%)	1% (18%×7%)	16% (18%×90%)
Dry, poor	3% (9%×30%)	0.9% (9%×10%)	6% (9%×60%)
Mesic, intermediate to rich	60 (73%×83%)	7% (73%×9%)	6% (73%×8%)

The right diagram of **Fig. 2** in the article shows the results when using the revised reference model (**Table 1** and **Fig. 3** in the article and **Appendix S1**). It emphasizes a prevalence (at least 50%) of old forests (≥150 years) due to a greater importance of non-stand-replacing disturbances and gap and cohort dynamics. Further, the three dynamics types occur in equal proportions (1/3 or 33.3% of each type) and are less strictly related to site type. Here, we adapt the revised reference model to the classification of young (0–109 years) and mid-aged (110–149 years) used in the analysis (see **Appendix S1**). The expected age-class distribution under natural conditions in the region studied is given by **Table S4**.

Table S4. The revised reference model with an age limit of 110 years between young and mid-age forests (see **Appendix S1** for explanation of model set-up and proportions of age classes). The dominance (at least 50%) of old forests (≥150 years), but also the equal shares (1/3 or 33.3%) of the three disturbance dynamics types comprise basic model settings and overall targets (highlighted figures in bold) derived from a review of current understanding of reference conditions.

	Young (0–109 years)	Mid (110-149 years)	Old (≥150 years)	Total
Gap dynamics, GD	6	5	23	**34**
Even-age dynamics, ED	25	3	5	**33**
Cohort dynamics, CD	6	5	22	**33**
Total	37	13	**50**	100

The targeted distribution of dynamics types across age classes (**Table S4**) can be attained by taking the available site-type distribution (**Table S1**) and their probable natural dynamics into account. In this example, gap dynamics are assumed to prevail on moist to wet site types (totally ca. 18%) and cohort dynamics on dry, poor site types (totally ca. 9%). Still, gap and cohort dynamics need to occur not only on these two major site types, but also on mesic, intermediate to rich site types to achieve the targeted shares of gap and cohort dynamics across age classes. The resulting distribution of dynamics types across age classes are shown in **Table S5**.

Table S5. The age-class distribution estimated using the revised reference model (used for the right diagram in **Fig. 2** in the article). The three disturbance dynamics types are distributed within each age class by taking the available site-type distribution (**Table S1**) and their probable natural dynamics into account; gap dynamics (GD) prevail on moist to wet site types and cohort dynamics (CD) on dry, poor site types. Still, gap and cohort dynamics need to occur not only on these two major site types, but also on mesic, intermediate to rich site types to attain the targeted shares across age classes (figures underlined). The targeted shares of even-aged dynamics (ED) across age classes are still attained on mesic, intermediate to rich site types.

	**Young (0–109 years)**	**Mid (110-149 years)**	**Old (≥150 years)**		**Total**
GD on wet to moist site types	3	2.5	12	18	**34**
GD on mesic, intermediate to rich site types	3	2.5	11	16	
ED on mesic, intermediate to rich site types	25	3	5		**33**
CD on mesic, intermediate to rich site types	4	4	16	24	**33**
CD on dry, poor site types	2	1.5	6	9	
Total	37	13	**50**		100

References

Aakala, T., T. Kuuluvainen, T. Wallenius, and H. Kauhanen. 2009. Contrasting patterns of tree mortality in late-successional *Picea abies* stands in two areas in northern Fennoscandia. *Journal of Vegetation Science* 20: 1016–1026.

Angelstam, P. and L. Andersson. 2001. Estimates of the needs for forest reserves in Sweden. Scandinavian Journal of Forest *Research* 16:S3: 38–51.

Angelstam, P. and T. Kuuluvainen. 2004. Boreal forest disturbance regimes, successional dynamics and landscape structures: a European perspective. *Ecological Bulletins* 51: 117–136.

Johnson, E.A. 1992. *Fire and vegetation dynamics: studies from the North American boreal forest.* Cambridge: Cambridge University Press.

Lilja, S., T. Wallenius, and T. Kuuluvainen. 2006. Structure and development of old *Picea abies* forests in northern boreal Fennoscandia. Écoscience 13: 181–192.

Statens Offentliga Utredningar (SOU) 1997: 97 och 1997: 98. ISBN 91-38-20641-2 (In Swedish).

Swedish National Forest Inventory, 2020. Unpublished data. The Department of Forest Resource Management. Swedish University of Agricultural Sciences, Umeå. http://www.slu.se/nfi.

Ylisirniö, A-l., R. Penttilä, H. Berglund, V. Hallikainen, L. Isaeva, H. Kauhanen, M. Koivula, and K. Mikkola, 2012. Dead wood and polypore diversity in natural post-fire succession forests and managed stands - Lessons for biodiversity management in boreal forests. *Forest Ecology and Management* 286: 16–27.
